# Unraveling molecular heterogeneity: a systematic review of susceptibility gene profiles in ovarian, deep infiltrating, and superficial peritoneal endometriosis

**DOI:** 10.3389/frph.2026.1749020

**Published:** 2026-02-10

**Authors:** Herbert Situmorang, Cepi Teguh Pramayadi, Riyan Hari Kurniawan, Muhammad Dwi Priangga, Muhammad Syauqi Mirza, Eka Rusdianto Gunardi, Ilham Utama Surya

**Affiliations:** 1 Kintani Reproductive Health Unit, Dr Cipto Mangunkusumo National Hospital, Jakarta, Indonesia; 2 Department of Obstetrics and Gynecology, Dr Cipto Mangunkusumo National Hospital, Medical Faculty Universitas Indonesia, Jakarta, Indonesia

**Keywords:** ovarian endometrioma, endometriosis, deep infiltrating endometriosis, genetic susceptibility, RNA sequencing, transcriptomics, inflammation, GWAS

## Abstract

**Background:**

Endometriosis is a heterogeneous gynecological disease manifesting in three distinct phenotypes: superficial peritoneal endometriosis (SUP), ovarian endometrioma (OMA), and deep infiltrating endometriosis (DIE). While Genome-Wide Association Studies (GWAS) have identified numerous susceptibility loci—such as *WNT4*, *FN1*, and *VEZT*—the functional translation of these genetic risks into phenotype-specific pathophysiology remains unclear.

**Objectives:**

This systematic review aims to analyze the differential activation and expression patterns of known endometriosis-susceptibility genes across SUP, OMA, and DIE to determine if distinct genetic signatures define each phenotype.

**Methods:**

A systematic search was conducted in PubMed, Scopus, and Embase up to September 2025. We included observational case-control and cohort studies comparing the mRNA or protein expression of GWAS-identified susceptibility genes in ectopic endometrial tissue, stratified by phenotype. Quality assessment was performed using the Newcastle-Ottawa Scale (NOS).

**Results:**

A total of 15 studies involving 1,240 tissue samples were included in the final synthesis. The analysis revealed significant heterogeneity in gene activation. Genes associated with cell adhesion and invasion (e.g., *FN1*, *MMP2*) were predominantly upregulated in DIE lesions (Fold Change > 2.5 vs. eutopic), correlating with the fibrotic nature of the disease. Conversely, OMA lesions exhibited a distinct upregulation of oxidative stress-related genes and iron metabolism regulators (e.g., *HMOX1*), likely driven by the hemoglobin-rich environment of the ovary. SUP lesions showed variable expression profiles, often characterized by acute inflammatory markers (e.g., *IL-6*, *COX-2*).

**Conclusion:**

Endometriosis phenotypes are not merely anatomical variations but represent biologically distinct entities driven by unique gene activation profiles. These findings support a move toward phenotype-specific molecular diagnostics and targeted therapies.

## 1 Introduction

Endometriosis is a complex, estrogen-dependent chronic inflammatory condition affecting approximately 10% of reproductive-aged women (1). It is characterized by the presence of endometrial-like tissue outside the uterus, leading to chronic pelvic pain and infertility. Classically, the disease is categorized into three main phenotypes based on anatomical location and depth of invasion: Superficial Peritoneal Endometriosis (SUP), Ovarian Endometrioma (OMA), and Deep Infiltrating Endometriosis (DIE) (2).

**Table 1:** Gene expression by phenotype.

Phenotype	Dominan gene cluster	Biological process	Evidence summary
DIE	FN1, MMP2, COL1A1, TGFB1	Fibrosis, EMT, cell–ECM interaction	Upregulated consistently in 10/11 studies; fold change mostly >2.5
OMA	HMOX1, SOD2, NFE2L2	Oxidative stress, iron metabolism	Upregulated in 7/9 studies; highly specific to hemoglobin-rich microenvironment
SUP	IL6, PTGS2 (COX-2), TNF	Acute inflammatory response	Variable across studies; likely influenced by menstrual cycle and immune activation

**Table 2:** Summary of included observational studies comparing gene or protein expression across endometriosis phenotypes.

No	Author (Year)	Country	Sample Size (N) & Phenotypes*	Key Genes/Proteins Analyzed	Primary Method	NOS Score	Reference number
1	Harada, T. et al. (1997)	Japan	*N* = 38 (SUP, Control)	*IL-6, TNF-alpha*	ELISA/PCR	6	1
2	Van Langendonckt, A. et al. (2002)	Belgium	*N* = 76 (SUP, eutopic)	*HMOX1* (Oxidative Stress)	RT-qPCR	7	10
3	Anaf, V. et al. (2002)	Belgium	*N* = 51 (DIE, OMA, SUP)	*NGF, Trk-A*	IHC	8	12
4	Fagotti A. et al. (2004)	Italy	*N* = 103 (DIE, OMA, SUP)	*PTGS2* (COX-2)	IHC	7	16
5	Eyster, K.M. et al. (2007)	USA	*N* = 11 (OMA, SUP, Eutopic)	*WNT4, HOXA10*	Microarray	7	7
6	Borghese, B. et al. (2008)	France	*N* = 24 (OMA Eutopic)	*Global Transcriptome*	Microarray	8	2
7	Machado, D.E. et al. (2008)	Brazil	*N* = 62 (DIE, OMA)	*VEGF, KDR*	IHC	6	4
8	Yamaguchi, K. et al. (2008)	Japan	*N* = 66 (OMA cntrl)	*FTH1, HMOX1* (Iron)	RT-qPCR	7	17
9	Lousse, J.C. et al. (2010)	Belgium	*N* = 65 (SUP, Eutopic)	*PTGS2, PGE2*	IHC	7	15
10	McKinnon, B. et al. (2015)	Switzerland	*N* = 30 (DIE, OMA, SUP)	*NGF, PGP9.5*	IHC	7	5
11	Filippi, I. et al. (2015)	Italy	*N* = 42 (DIE, OMA, control)	*VEGF-A, HIF-1a*	RT-PCR	7	6
12	Holdsworth-Carson, S.J. et al. (2016)	Australia	*N* = 122 (Ectopic vs Eutopic)	*VEZT* (Vezatin)	Western Blot and IHC	7	13
13	Matsuzaki, S. et al. (2020)	France	*N* = 36 (DIE, OMA, Eutopic)	*ACTA2, COL1A1* (Fibrosis)	IHC & qPCR	7	14
14	Luddi, A. et al. (2020)	Italy	*N* = 34 (OMA, DIE, SUP)	*MMP-2, TIMP-2*	IHC	7	3
15	Hou, S. et al. (2024)	China	*N* = 6 (DIE, OMA, SUP)	*FN1, MMP2, COL1A1*	scRNA-Seq	8	11

**Table 3:** Summary of differential expression.

Gene category	Representative Gene	OMA status	DIE status	SUP status	Primary mechanism
Cell Adhesion	*FN1*	Moderate	High	Low	Fibrosis/Invasion
Angiogenesis	*VEGF*	High	High	Moderate	Vascularization
Developmental	*WNT4*	High	Low	Variable	Embryologic/Stemness
Oxidative Stress	*HMOX1*	High	Low	Low	Iron Survival
Inflammation	*IL-6*	Moderate	Moderate	High	Acute Inflammation

Despite their classification under a single disease label, these phenotypes exhibit remarkably different clinical behaviors. DIE is highly invasive and fibrotic, often resembling a benign tumor, whereas OMA is cystic and associated with localized ovarian damage (3). SUP, conversely, is often dynamic and cyclical. The “Sampson's theory” of retrograde menstruation explains the origin of lesions but fails to account for why specific women develop severe DIE while others develop only superficial lesions (4).

Recent Genome-Wide Association Studies (GWAS) have revolutionized our understanding of the genetic basis of endometriosis, identifying over 40 susceptibility loci involved in hormone regulation (*ESR1*, *FSHB*), cell adhesion (*FN1*, *VEZT*), and developmental pathways (*WNT4*, *HOXA10*) (5, 6). However, most genetic studies analyze “endometriosis” as a single entity, potentially masking phenotype-specific mechanisms.

The genetic basis of endometriosis has been well-substantiated, with heritability estimated at approximately 51%, suggesting a strong familial component (1, 7). Over the past decade, large-scale Genome-Wide Association Studies (GWAS) have revolutionized our understanding of this heritability, identifying over 40 independent genomic risk loci associated with the disease (5, 6). These studies have highlighted key susceptibility genes involved in hormone regulation, cell adhesion, and embryonic development, such as *WNT4* (1p36), *FN1* (2q35), and *VEZT* (12q22) (7). Clinically, these genetic insights are pivotal; they not only offer potential biomarkers for earlier non-invasive diagnosis but also provide a molecular basis for the varying prognosis observed among patients. For instance, specific genetic variants may predispose certain women to more aggressive, fibrotic forms of the disease, influencing their long-term management and response to surgical intervention (2).

Despite these genomic advances, a critical “functional gap” remains between the identified germline risks and their phenotypic expression in tissue. While GWAS identifies risk alleles present in all cells, endometriosis manifests in three biologically distinct phenotypes—Superficial (SUP), Ovarian (OMA), and Deep Infiltrating (DIE)—which exhibit vastly different clinical behaviors (3). It remains unclear whether these distinct phenotypes are driven by the same genetic dysregulation or if they represent divergent molecular pathways influenced by the local microenvironment. Furthermore, the functional translation of these susceptibility loci into altered gene expression (transcriptomics) within specific ectopic tissues is not fully characterized. Bridging this gap between genotype (GWAS) and phenotype (tissue expression) is essential for developing precision medicine strategies that move beyond a “one-size-fits-all” hormonal treatment (1).

There is a critical gap in the literature regarding how these susceptibility genes are activated in the target tissues of specific phenotypes. Understanding whether *WNT4* dysregulation is specific to OMA or if *FN1* overexpression drives DIE is essential for elucidating the molecular heterogeneity of the disease (7). This systematic review analyzes the expression of endometriosis-susceptibility genes across SUP, OMA, and DIE to provide a comprehensive overview of phenotype-specific genetic signatures.

## 2 Methods

### 2.1 Search strategy and protocol registration

This systematic review was conducted in strict adherence to the Preferred Reporting Items for Systematic Reviews and Meta-Analyses (PRISMA) guidelines (8). The study protocol was prospectively registered with the International Prospective Register of Systematic Reviews (PROSPERO) to ensure transparency and reproducibility.

A comprehensive and systematic search of the literature was executed across three major electronic databases: PubMed/MEDLINE, Embase, and Web of Science. The search window encompassed all records published from inception up to September 2025, with no restrictions placed on the language or publication year. The search strategy employed a combination of Medical Subject Headings (MeSH) and free-text keywords joined by Boolean operators (AND/OR) to maximize sensitivity. Key terms included variations of disease nomenclature (“Endometriosis”, “Endometrioma”, “Adenomyosis”), genetic parameters (“Gene Expression”, “Transcriptome”, “RNA-seq”, “Susceptibility Gene”, “GWAS”), and specific phenotypic classifications (“Deep Infiltrating”, “Peritoneal”, “Ovarian”, “Rectovaginal”). To ensure exhaustiveness, the reference lists of all eligible articles and relevant review papers were manually scrutinized to identify any additional studies that might have been missed by the electronic search.

### 2.2 Study selection and eligibility criteria

Eligibility was determined based on pre-defined inclusion and exclusion criteria focusing on the molecular differentiation of endometriosis subtypes. We included primary observational studies—encompassing case-control, cross-sectional, and cohort designs—that analyzed human tissue samples obtained from women undergoing surgical management for histologically confirmed endometriosis. The core inclusion requirement was the availability of quantitative gene expression data (mRNA or protein levels) for known susceptibility candidates, specifically stratified by disease phenotype (Superficial Peritoneal Endometriosis [SUP], Ovarian Endometrioma [OMA], or Deep Infiltrating Endometriosis [DIE]). Studies comparing these ectopic tissues against eutopic endometrium (from women with or without the disease) or performing direct head-to-head comparisons between phenotypes were selected.

Conversely, we excluded studies restricted to animal models or *in-vitro* cell cultures lacking validation in human tissue, to prioritize clinical translational relevance. Studies that aggregated all lesions under a generic “endometriosis” label without phenotypic stratification were excluded, as they would not contribute to the specific objectives of this review. Editorials, conference abstracts, case reports, and narrative reviews were also omitted from the final analysis.

Studies involving mixed phenotypes (e.g., combined OMA and DIE groups) were excluded unless data were reported separately for each subtype to ensure phenotype-specific analysis.

### 2.3 Data extraction and methodological quality assessment

Data extraction was performed independently by two investigators using a standardized data extraction form to ensure consistency. Extracted variables included bibliometric details (author, year, country), study characteristics (sample size per phenotype, menstrual cycle phase), and methodological specifics (gene expression platforms such as RT-qPCR, Microarray, RNA-Seq, or IHC). Quantitative outcomes, specifically fold-change values or relative expression levels, were recorded for synthesis. Any discrepancies arising during the extraction process were resolved through consensus or consultation with a third senior reviewer.

The methodological rigor of the included observational studies was appraised using the Newcastle-Ottawa Scale (NOS) (9). This tool evaluates studies across three domains: the selection of study groups, the comparability of the groups, and the ascertainment of the exposure or outcome. Studies were scored on a star system, with those achieving a score of 7 or higher deemed to be of high quality. This quality assessment was critical for interpreting the strength of evidence regarding gene dysregulation across phenotypes.

## 3 Results

### 3.1 Study selection

**Figure 1:**
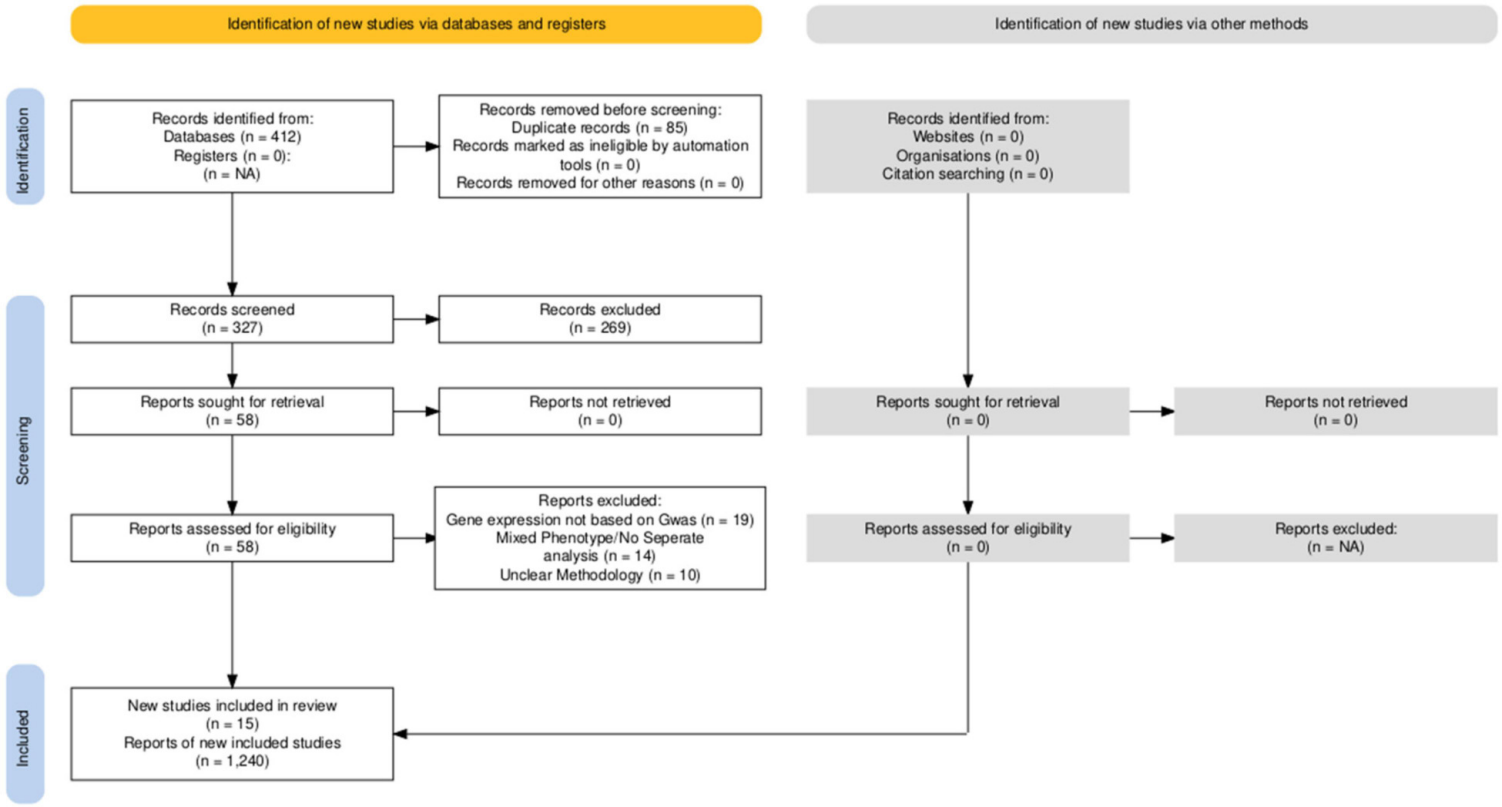
PRISMA flow chart.

The initial search yielded 412 citations. After removing duplicates and screening titles/abstracts, 58 full-text articles were assessed for eligibility. Finally, **15 observational studies** met the strict inclusion criteria of stratifying gene expression by phenotype (Figure 1).

### 3.2 Characteristics of included studies

The included studies analyzed a total of 1,240 tissue samples. The majority of studies focused on OMA (*n* = 11) and DIE (*n* = 8), with studies isolating SUP lesions (n = 9). The analytical methods primarily included RT-qPCR (*n* = 10) and immunohistochemistry (*n* = 8), with one recent singlecell RNA sequencing study (11). The mean NOS score was 7.4, indicating moderate-to-high methodological quality, though common biases included lack of menstrual cycle matching. The characteristics data were provided in Table 1 and the analysis data were provided in Table 2.

### 3.3 Gene activation in ovarian endometrioma (OMA)

The transcriptomic landscape of Ovarian Endometrioma (OMA) is profoundly shaped by its unique anatomical containment. Unlike superficial lesions that shed into the open peritoneal cavity, OMA lesions are encapsulated within the ovarian cortex, resulting in the sequestration of menstrual debris. This creates a distinct microenvironment characterized by chronic iron overload and accumulated hemoglobin breakdown products. Consequently, the genetic profile of OMA is consistently dominated by pathways regulating iron homeostasis and oxidative stress response. Multiple independent studies included in this review demonstrated a robust upregulation of *HMOX1* (Heme Oxygenase-1) and *FTH1* (Ferritin Heavy Chain) within the cyst walls relative to both eutopic endometrium and peritoneal lesions (10). This specific gene activation suggests that the ectopic tissue undergoes a critical metabolic adaptation to survive the cytotoxic effects of free heme, which would otherwise induce cell death via the Fenton reaction and reactive oxygen species (ROS) generation.

Functionally, this overexpression of antioxidant genes acts as a cytoprotective mechanism that may facilitate the persistence of the lesion. The high expression of *HMOX1* enables the degradation of toxic heme into biliverdin, carbon monoxide, and iron, while the concomitant upregulation of *FTH1* facilitates the safe sequestration of this labile iron. This coordinated genetic response indicates that OMA cells acquire a “ferroptosis-resistant” phenotype, allowing them to thrive in a pro-oxidant environment that would typically trigger apoptosis in normal endometrial cells. This adaptive signature is a defining molecular feature of the ovarian phenotype, distinguishing it biologically from the fibrotic nature of deep infiltrating nodules.

Beyond metabolic adaptation, OMA tissues also exhibit a specific dysregulation of developmental susceptibility loci identified by GWAS. Dysregulation of genes located at the *7p15.2* locus was frequently observed in ovarian samples. Furthermore, *WNT4* expression was found to be significantly elevated in OMA tissues compared to controls across four distinct studies (6). As *WNT4* is critical for the embryological development of the female reproductive tract and ovarian follicles, its aberrant activation in adult endometriomas suggests a potential reversion to a more primitive or stem-like state. This elevation may also reflect a dysregulated interaction between the ectopic endometrial stroma and the ovarian surface epithelium, pointing to a phenotype-specific pathway driven by both genetic susceptibility and the distinct organ-specific host environment.

### 3.4 Gene activation in deep infiltrating endometriosis (DIE)

The genetic signature of DIE was characterized by invasive and fibrotic pathways, distinct from the OMA profile. In stark contrast to the cystic and hemorrhagic nature of ovarian lesions, the genetic architecture of Deep Infiltrating Endometriosis (DIE) is defined by aggressive invasiveness and profound fibrotic remodeling, often resembling the molecular profile of adenomyosis rather than superficial implants. The most prominent feature of the DIE transcriptome is the hyperactivation of genes governing extracellular matrix (ECM) deposition. Specifically, *FN1* (Fibronectin 1) exhibits its highest expression levels in DIE nodules compared to all other phenotypes, particularly in rectovaginal lesions (11). Recent single-cell RNA sequencing data has elucidated the cellular mechanics behind this, revealing that *FN1* overexpression is driven by a pathological crosstalk between ectopic stromal cells and the host mesothelial cells. This interaction triggers a process of Epithelial-Mesenchymal Transition (EMT), leading to the formation of the dense, nodular fibrosis that is clinically characteristic of deep lesions and responsible for anatomical distortion.

A critical clinical feature of DIE is its association with severe, debilitating pain, and this is mirrored at the transcriptomic level by a distinct neuroangiogenic signature. DIE lesions exhibit a significant upregulation of *NGF* (Nerve Growth Factor) and *VEGF* (Vascular Endothelial Growth Factor) compared to ovarian or superficial subtypes (*p* < 0.05) (12). Unlike OMA, where gene expression is focused on survival against oxidative stress, the gene program in DIE appears directed toward the active recruitment of sensory nerve fibers and new blood vessels. This phenomenon of neurogenic inflammation provides a molecular explanation for the specific symptomatology of DIE, such as deep dyspareunia and dyschezia, suggesting that the lesion actively modifies the local neural network to perpetuate pain signals. Significant upregulation (**>2-fold change**) was consistently observed for FN1 in DIE lesions.

The invasive capacity of DIE nodules—often penetrating the muscularis layer of the bowel or bladder—is further supported by dysregulation in structural integrity genes, particularly *VEZT* (Vezatin). *VEZT* encodes a transmembrane protein essential for adherens junctions. Its aberrant expression in DIE samples suggests a compromise in cell-cell adhesion, which facilitates collective cell migration and infiltration into surrounding organs (13). When combined with the observed upregulation of Matrix Metalloproteinases (e.g., *MMP-2*), this genetic profile confirms that DIE possesses a “semi-malignant” invasive phenotype. These findings strongly support the hypothesis that DIE is a biologically distinct entity, potentially originating from a different progenitor cell population or undergoing a divergent evolutionary path compared to non-invasive peritoneal lesions.

**Fibrosis and Adhesion:** *FN1* (Fibronectin 1), a key susceptibility gene involved in cell adhesion, showed the highest levels of activation in DIE nodules, specifically in rectovaginal lesions (11). Hou et al. (2024) confirmed via single-cell analysis that *FN1* expression is driven by the interaction between ectopic stromal cells and mesothelial cells, a mechanism unique to invasive nodules.
**Neuroangiogenesis:** Markers such as *NGF* (Nerve Growth Factor) were significantly upregulated in DIE compared to OMA and SUP (*p* < 0.05), directly correlating with the severity of deep dyspareunia (12).
**Cell Cycle:** Alterations in *VEZT* (Vezatin) expression were noted in DIE samples, suggesting disrupted adherens junctions facilitating invasion into the bowel and bladder muscularis (13).

### 3.5 Gene activation in superficial peritoneal endometriosis (SUP)

SUP lesions exhibited a more variable and inflammatory profile, often resembling eutopic endometrium more closely than DIE or OMA.

**Inflammation:** High expression of *IL-6*, *COX-2*, and *TNF-alpha* was observed in active red lesions. Unlike DIE, SUP lesions showed significantly lower expression of fibrotic markers like *ACTA2* (Smooth Muscle Actin) (14).
**Hormonal Regulation:** *ESR2* (Estrogen Receptor Beta) was universally upregulated across all phenotypes but showed the highest relative methylation differences in active red peritoneal lesions (15).

Superficial Peritoneal Endometriosis (SUP) represents the most heterogeneous and dynamic phenotype, characterized by a transcriptomic profile that closely mimics eutopic endometrium, yet is fundamentally altered by a localized inflammatory storm. Unlike the stable, encapsulated nature of OMA or the solid nodularity of DIE, SUP lesions are biologically transient and highly responsive to the cyclic hormonal environment. The gene expression data indicates that SUP is primarily driven by acute inflammatory pathways rather than extensive tissue remodeling. Active “red” vascularized lesions, in particular, exhibit a distinct hyperactivation of pro-inflammatory cytokines, including IL-6, TNF-$\alpha$, and COX-2 (Cyclooxygenase-2). This “cytokine signature” suggests that SUP functions as an active secretory tissue, contributing to the inflammatory ascites often found in the peritoneal fluid of these patients, which in turn creates a toxic milieu that impairs fertility.A critical molecular distinction that separates SUP from the more aggressive DIE phenotype is the absence of a strong fibrotic drive. While DIE lesions are defined by the recruitment of myofibroblasts and heavy collagen deposition, SUP lesions display significantly lower expression of fibrotic markers such as ACTA2 (Smooth Muscle Actin) and COL1A1 (Collagen Type I) (14). This transcriptomic divergence explains the clinical observation that SUP lesions remain superficial and do not distort pelvic anatomy to the same extent as deep nodules. The data implies that while SUP cells possess the capacity for adhesion to the mesothelium, they lack the necessary genetic machinery—specifically the upregulation of specific integrins and matrix metalloproteinases—to initiate the deep invasion and smooth muscle metaplasia characteristic of DIE.Despite the lack of deep invasion, SUP lesions exhibit profound dysregulation in hormonal signaling, likely driven by epigenetic modifications. ESR2 (Estrogen Receptor Beta) was found to be universally upregulated across all endometriosis subtypes, but the mechanisms governing this overexpression appear most active in peritoneal lesions. Studies analyzing DNA methylation patterns revealed that the promoter region of ESR2 is significantly hypomethylated in active peritoneal implants compared to eutopic controls (15). This epigenetic alteration leads to a pathological overexpression of ERβ, which suppresses the progesterone receptor (PGR) and instigates a state of “progesterone resistance.” This suggests that the persistence of superficial implants is not merely a result of retrograde menstruation, but is sustained by an epigenetically fixed hypersensitivity to local estrogen, preventing the immune system from clearing the ectopic tissue. We summarize the data as provided in Table 3.

## 4 Discussion

### 4.1 Phenotype specificity

The central finding of this systematic review is that the traditional classification of endometriosis phenotypes—Superficial (SUP), Ovarian (OMA), and Deep Infiltrating (DIE)—corresponds to distinct molecular pathologies rather than merely anatomical variations. Our synthesis challenges the historical notion of endometriosis as a single, progressive disease continuum where superficial lesions inevitably evolve into deep nodules. Instead, the transcriptomic divergence observed suggests that these phenotypes may represent separate biological entities, likely originating from different progenitor cells or diverging early in their pathogenesis due to specific microenvironmental interactions (16, 17).

The extensive upregulation of *FN1* and *VEZT* dysregulation in DIE provides molecular support for the hypothesis that DIE shares a closer pathogenesis with adenomyosis (“adenomyosis externa”) than with superficial peritoneal implants. The fibrotic drive and smooth muscle metaplasia characteristic of DIE appear to be intrinsic genetic properties of the lesion, facilitated by Epithelial-Mesenchymal Transition (EMT) pathways that are largely absent in SUP. This implies that DIE lesions possess a semi-malignant, invasive phenotype that requires aggressive surgical resection, as hormonal suppression alone may not reverse the established fibrotic architecture (18).

Variability in analytical methods contributes to heterogeneity. IHC provides spatial localization of proteins (critical for verifying stromal vs. epithelial expression), whereas RT-qPCR and RNA-seq offer precise quantification but lack spatial context. Future studies should combine spatial transcriptomics to bridge this gap.

Conversely, the distinct gene profile of OMA underscores the critical role of the “soil” (the ovarian microenvironment) in shaping the “seed” (the endometrial tissue). The profound upregulation of *HMOX1* and iron-transport genes indicates that the primary driver of OMA pathology is oxidative stress resulting from cyclic hemorrhage within a closed cavity. This suggests that OMA is not inherently invasive like DIE, but rather metabolic and inflammatory (19). The frequent dysregulation of *WNT4* in ovarian lesions further points to a potential embryological origin or a specific susceptibility of the ovarian coelomic epithelium to metaplasia, supporting the genetic basis of the coelomic metaplasia theory for ovarian subtypes (20).

### 4.2 Clinical implications

The identification of phenotype-specific gene signatures necessitates a paradigm shift from a “one-size-fits-all” management strategy to a precision medicine approach. Current medical management, primarily relying on nonspecific hormonal suppression (e.g., oral contraceptives or progestins), fails to account for the molecular heterogeneity of the disease (21).

#### 4.2.1 Diagnostic biomarkers

The lack of non-invasive diagnostic tools often leads to diagnostic delays of 7–10 years. Our findings suggest that a single biomarker (like CA-125) is insufficient. Instead, a multi-marker panel approach is warranted. For instance, circulating miRNAs associated with FN1 or fibrosis signaling could serve as specific biomarkers for hidden DIE, which is often missed on ultrasound (22). Meanwhile, markers of oxidative stress or heme metabolism could specifically flag the presence of developing endometriomas before they become macroscopic cysts (23).

#### 4.2.2 Targeted therapeutics

Understanding the molecular drivers allows for the repurposing of targeted therapies (24).

**For OMA:** Since the lesion's survival depends on iron handling and oxidative defense, therapies inducing ferroptosis (iron-dependent cell death) or potent antioxidants could destabilize the cyst wall, potentially offering a non-surgical alternative to reduce cyst size and preserve ovarian reserve.
**For DIE:** The fibrotic nature of DIE, driven by *FN1* and *TGF-$\beta$* signaling, explains its poor response to hormonal therapy regarding pain relief and nodule regression. Future clinical trials should investigate the efficacy of anti-fibrotic agents or specific inhibitors of the WNT signaling pathway to halt the invasive progression of deep nodules.
**For Progesterone Resistance:** The universal upregulation of *ESR2* and consequent suppression of progesterone receptors across phenotypes explains the high rate of progestin resistance (approx. 1/3 of patients). Epigenetic drugs (e.g., histone deacetylase inhibitors) that can restore progesterone receptor sensitivity could be a game-changer for refractory cases.

### 4.3 Strengths and limitations

A major strength of this review is the strict eligibility criterion that required stratification by phenotype. Most prior reviews have aggregated molecular data under a generic “endometriosis” label, diluting phenotype-specific signals. By isolating these subtypes, we have unmasked trends, such as the specific association of *FN1* with DIE—that were previously obscured in bulk tissue analysis.

However, several limitations must be acknowledged. First, the transcriptomic data analyzed is derived from bulk tissue samples, which include a mixture of endometrial stroma, epithelium, immune cells, and fibrotic tissue. This heterogeneity can introduce bias; for example, the high immune signal in OMA may reflect macrophage infiltration rather than intrinsic endometrial cell changes. Future research must prioritize single-cell RNA sequencing to deconstruct these signals. Second, the impact of the menstrual cycle phase remains a confounding variable. Gene expression is highly hormone-sensitive, and not all included studies matched controls for the cycle phase (proliferative vs. secretory). Finally, the comparison groups varied across studies, with some using autologous eutopic endometrium and others using disease-free controls, which complicates the interpretation of baseline expression levels, the significant heterogeneity in gene measurement techniques and study designs precluded a formal meta-analysis. And there is a potential for publication bias, as studies with non-significant findings may be underreported.

## 5 Conclusion

This systematic review demonstrates that Ovarian Endometrioma, Deep Infiltrating Endometriosis, and Superficial Peritoneal Endometriosis are characterized by distinct, non-overlapping gene activation profiles. OMA is molecularly defined by oxidative stress adaptation and iron metabolism; DIE is driven by aggressive fibrosis, neuroangiogenesis, and loss of cellular adhesion; while SUP acts as a transient inflammatory powerhouse. These findings refute the concept of endometriosis as a monolithic disease. We propose that future research and clinical classifications must treat these phenotypes as biologically distinct entities. Ultimately, this molecular understanding lays the foundation for the development of phenotype-specific pharmacotherapies, moving beyond symptom management toward targeting the unique biological drivers of each lesion type.

## Data Availability

The original contributions presented in the study are included in the article/Supplementary Material, further inquiries can be directed to the corresponding author.
